# Supplementary material for: Antibody-controlled actuation of DNA-based molecular circuits
Source: Nat Commun. 2017 Feb 17;8:14473. doi: 10.1038/ncomms14473 (PMC5321729; doi:10.1038/ncomms14473)
Supplement: Supplementary Information — Supplementary Figures, Supplementary Tables, Supplementary Methods and Supplementary References. [file ncomms14473-s1.pdf]

Supplementary Methods

Oligonucleotide sequences

Supplementary Table 1 | oligonucleotide sequences used for toehold screening and molecular computing

**Name (domains)**[a]	**Sequence**[b]	**Experiments**
$\mathbf{B_x}$ (1-2-3)	5'-TGGAGA CGTAGGGTATTGAATGTGCTGTAG-NH_2-3'	Toehold screening
$\mathbf{I_x}$ (3*-2*)	5'-CTACAGCACATTCAATACCCTACG-NH_2-3'	
$\mathbf{O_{x0}}$ (2*-1*)	5'-CTACAGCACATTCAATACCCTACG TCTCCA-3'	
$\mathbf{O_{x1}}$ (2*-1*)	5'-TACAGCACATTCAATACCCTACG TCTCCA-3'	
$\mathbf{O_{x2}}$ (2*-1*)	5'-ACAGCACATTCAATACCCTACG TCTCCA-3'	
$\mathbf{O_{x3}}$ (2*-1*)	5'-CAGCACATTCAATACCCTACG TCTCCA-3'	
$\mathbf{O_{x4}}$ (2*-1*)	5'-AGCACATTCAATACCCTACG TCTCCA-3'	
$\mathbf{O_{x5}}$ (2*-1*)	5'-GCACATTCAATACCCTACG TCTCCA-3'	
$\mathbf{O_{x6}}$ (2*-1*)	5'-CACATTCAATACCCTACG TCTCCA-3'	
$\mathbf{F_x}$ (2*)	5'-FAM-CATTCAATACCCTACG-3'	
$\mathbf{Q_x}$ (1-2)	5'-TGGAGA CGTAGGGTATTGAATG-IowaBlackFQ-3'	
$\mathbf{B_y}$ (4-5-6)	5'-GCAATC GACCTGCGTAAGTCTCTTCACCTC-NH_2-3'	Orthogonal sequences
$\mathbf{I_y}$ (6*-5*)	5'-GAGGTGAAGAGACTTACGCAGGTC-NH_2-3'	
$\mathbf{O_{y3}}$ (5*-4*)	5'-GTGAAGAGACTTACGCAGGTC GATTGC-3'	
$\mathbf{F_y}$ (5*)	5'-TEX615-GAGACTTACGCAGGTC-3'	
$\mathbf{Q_y}$ (4-5)	5'-GCAATC GACCTGCGTAAGTCTC-IowaBlackRQ-3'	
$\mathbf{O_{x\text{-}NOR}}$ (2*-1*)	5'-CAGCACATTCAATACCCTACG TCTCCA-IowaBlackFQ-3'	NOR-gate
$\mathbf{F_{NOR}}$ (1-2)	5'-FAM-TGGAGA CGTAGGGTATTGAATG-3'	
$\mathbf{H_{NOR}}$ (2*)	5'-CATTCAATACCCTACG-3'	
$\mathbf{F_{AND}}$ (5*)	5'-FAM-AAGAGACTTACGCAGGTC -3'	AND-gate
$\mathbf{Q_{AND}}$ (1-2-4-5)	5'-TGGAGACG TAGGGTATTGAATGTGCTG GCAATC GACCTGCGTAAGTCTCTT-IowaBlackFQ-3'	
$\mathbf{H_{x\text{-}(N)AND}}$ (4*-2*)	5'-GATTGC CAGCACATTCAATACCCTA-3'	
$\mathbf{O_{y\text{-}NAND}}$ (5*-4*)	5'-GTGAAGAGACTTACGCAGGTC GATTGC-IowaBlackRQ-3'	NAND-gate
$\mathbf{F_{NAND}}$ (1-2-4-5)	5'-TGGAGACG TAGGGTATTGAATGTGCTG-cy5-GCAATCGACCTGCGTAAGTCTCTT-3'	
$\mathbf{H_{y\text{-}NAND}}$ (5*)	5'-AAGAGACTTACGCAGGTC-3'	

[a] Numbers represent individual oligonucleotide, domains with * indicating complementary sequences.
[b] Colors represent individual oligonucleotide domains.

Supplementary Table 2 | Oligonucleotide sequences used for fully autonomous molecular circuits

**Name (domains)**[a]	**Sequence**[b]	**Experiments**
$\mathbf{B_{Dz}}$ (1-2-3-4)	5'-GATGTA TCTTAGTT TTCGACCGGCTCG TAG-NH_2-3'	DNAzyme actuation
$\mathbf{I_{Dz}}$ (4*-3*-2*)	5'-CTA CGAGCCGGTCGAA AACTAAGA-NH_2-3'	
$\mathbf{O_{Dz}}$ (3*-2*-1*)	5'-CGAGCCGGTCGAA AACTAAGA TACATC-3'	
**Inh** (1-2-3)	5'-GATGTA TCTTAGTT TTCGACCGGCTCG-3'	
**Dz** (5*-3*-2*) [c]	5'-GAACTATCTC CGAGCCGGTCGAA AACTAAGA-3'	
**S** (2-5)	5'-FAM-TCTTAGTT rAGGATAGTTCA-TAMRA-3'	
$\mathbf{B_{PPI}}$ (1-2-3)	5'-TGGAGA TAGACAGTTTCATCGGTGACA TAG-NH_2-3'	Enzyme actuation
$\mathbf{I_{PPI}}$ (3*-2*)	5'-CTATGTCACCGATGAAACTGTCTA-NH_2-3'	
$\mathbf{O_{PPI}}$ (2*-1*)	5'-TGTCACCGATGAAACTGTCTATCTCCA-3'	
$\mathbf{T_{PPI}}$ (3-linker-1-2)	5'-TTCCTCTACCACCTACATCAC CACAACACATTCAACACGCAACCA TGGAGA TAGACAGTTTCATCGGTGACA-3'	
**βLacE104D** (2*)	5'-TGTCACCGATGAAACTGTCTA-NH_2-3'	
**BLIP** (3*)	5'-H_2N-GTGATGTAGGTGGTAGAGGAA-3'	

[a] Numbers represent individual oligonucleotide domains with * indicating complementary sequences.
[b] Colors represent individual oligonucleotide domains.
[c] Underlined sequence denote the DNAzyme's catalytic core.

All oligonucleotides were dissolved to approximately 100 µM concentrations in TE buffer (10 mM Tris-HCl, 1 mM EDTA, pH 8.0) and quantified by UV-VIS (NanoDrop 1000, Thermo Scientific) using the extinction coefficients at 260 nm calculated with the Integrated DNA Technologies DNA UV spectrum predictor tool.[1] Subsequently, the oligonucleotides were further diluted, yielding stock solutions of 50 µM and stored at -30 °C.

Chemicals

All oligonucleotides were purchased HPLC purified from Integrated DNA Technologies. Sulfo-SMCC and anti-HA epitope tag antibody (clone: 2-2.2.14) were purchased from ThermoFisher Scientific. Anti-HIV-1 p17 antibody (clone:32/1.24.89) was obtained from ZeptoMetrix. All other reagents and solvents were obtained from commercial sources and used without further purification. Antibodies were quantified by UV-VIS (NanoDrop 1000, Thermo Scientific) using the extinction coefficient at 280 nm of 210,000 M^{-1} cm^{-1}, aliquoted and stored at -30 °C.

Thermal annealing of reporter duplexes

Reporter duplexes were obtained by mixing the individual oligonucleotide components together in equimolar amounts in TE/Mg^{2+} buffer (10 mM Tris-HCl, 1 mM EDTA, 12.5 mM $MgCl_2$, pH 8.0) to a final concentration of 5 µM and annealed in a thermocycler (BioRad) from 90 °C to 10 °C in 1 h and subsequently stored until use at 4 °C. AND and NAND gate reporter duplexes were further purified by native PAGE (10%). Gels were run in 1x TBE/Mg^{2+} buffer (89 mM Tris-HCl, 89 mM boric acid, 2 mM EDTA, 12.5 mM $MgCl_2$ pH 8.5) for 90 minutes at 125 V and stained with SYBR gold (Thermo Scientific). The band corresponding to the desired duplex was cut out and incubated in TE/Mg^{2+} buffer for 24 hours. Subsequently, the solution was extracted using Freeze 'N Squeeze DNA gel extraction spin column (BioRad) filtration and subsequently concentrated by spin filtration.

Peptide epitope synthesis and purification

Peptides were synthesized on an automated peptide synthesizer (Intavis, Multipep RSi) following standard Fmoc peptide synthesis on Rink Amide MBHA resin at a 200 µmol scale. The peptides were cleaved from the resin by a mixture of TFA / TIS / H_2O / EDT (92.5: 2.5: 2.5: 2.5% v/v) for 3 h at room temperature under continuous shaking and precipitated in ether at -30°C. The precipitated peptides were dissolved in a mixture of H_2O/acetonitrile + 0.1% TFA and purified by preparative reversed phase HPLC-MS on a Shimadzu LC-9A HPLC system using a VYDAC protein & peptide C18 column with a gradient of 20-40% acetonitrile in H_2O in 20 minutes. ESI-MS spectra were recorded using a Quadrupole Electrospray Ionization Mass Spectrometer in positive mode (API-150EX, Applied Biosystems) HA-epitope: YPYDVPDYA-GGG-C, calculated mass: 1375.46 Da; observed mass: 1375.34 Da. HIV1-p17-epitope: ELDRWEKIRLRP-GGG-CG, calculated mass: 1941.25 Da; observed mass: 1941.75 Da.

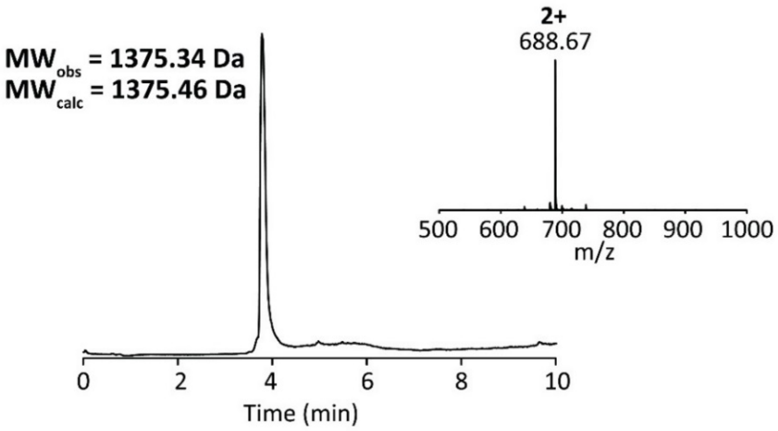

Supplementary Figure 1 | HPLC chromatogram and m/z spectrum of purified HA-epitope peptide (YPYDVPDYA-GGG-C)

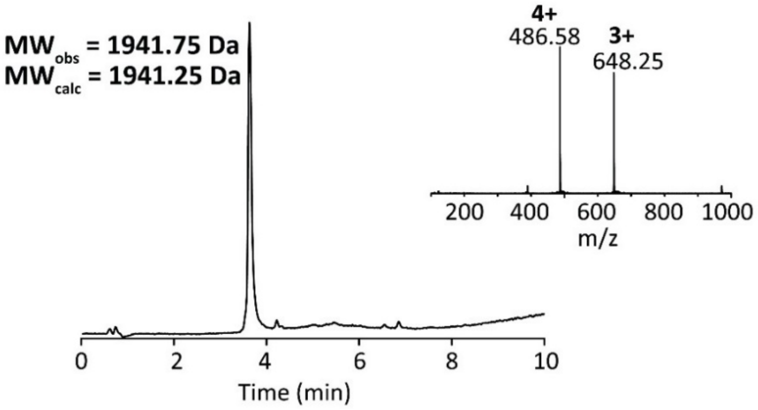

Supplementary Figure 2 | HPLC chromatogram and m/z spectrum of purified HIV-epitope peptide (ELDRWEKIRLRP-GGG-CG)

Peptide-Oligonucleotide Conjugates (POCs) synthesis and purification

Oligonucleotides modified with a 3'-primary amine were dissolved in PBS (100 mM NaPi, 150 mM NaCl, pH 7.2) and mixed with 20 equivalents of Sulfo-SMCC (Thermo Fisher Scientific, 2 mg no-weigh format, dissolved in DMSO prior to use) to a final concentration of 1 mM in 50% DMSO for 2 h at room temperature. The maleimide-functionalized oligonucleotides were isolated using ethanol precipitation by the addition of 10 % (v/v) 5 M NaCl and 300 % (v/v) ice cold ethanol and subsequently stored at -30 °C for 1 h. The precipitated oligonucleotides were pelleted by centrifugation for 15 min. at 14,000 rpm at 4 °C. After washing the pellet with ice cold 75 % ethanol the pellet was dried under vacuum. Prior to use, the maleimide-activated oligonucleotides were dissolved in Phosphate buffer (100 mM NaPi, pH 7.0) and mixed with 10 equivalents of peptide epitope (dissolved in Phosphate buffer) to a final concentration of 1 mM for 2 h at room temperature. Finally, the obtained POCs were purified by reversed-phase HPLC on a GraceAlpha C18 (250 x 4.6 mm) column using a gradient of 5 – 50% acetonitrile in 100 mM triethylammonium acetate (TEAA, pH 7.0) and lyophillized three times to a white powder. Purified POCs were analyzed using mass spectrometry by flow injection analysis on a LCQ Fleet (Thermo Finnigan) ion-trap mass spectrometer in negative mode. 5 µL of POC solution, dissolved to 10 µM in 1:1 isopropanol/water + 1% triethylamine (pH 10) was directly injected. Deconvoluted m/z spectra were obtained with MaxEnt software (Supplementary Figures 3 and 4, Supplementary Table 3).

Supplementary Table 3 | Composition of synthesized POCs with corresponding calculated and observed masses

Name	Oligonucleotide	Peptide	Calculated mass (Da)	Observed mass (Da)
**B_x-HA**	B_x	HA	11202.29	11202.26
**I_x-HA**	I_x	HA	9030.96	9030.79
**B_x-HIV**	B_x	HIV	11767.75	11766.13
**I_x-HIV**	I_x	HIV	9596.42	9595.43
**B_y-HA**	B_y	HA	10882.23	10882.84
**I_y-HA**	I_y	HA	9271.99	9271.24
**B_{dz}-HA**	B_{dz}	HA	11024.24	11024.06
**I_{dz}-HA**	I_{dz}	HA	9184.99	9184.51
**B_{PPI}-HA**	B_{PPI}	HA	11115.29	11115.04
**I_{PPI}-HA**	I_{PPI}	HA	9116.96	9116.27

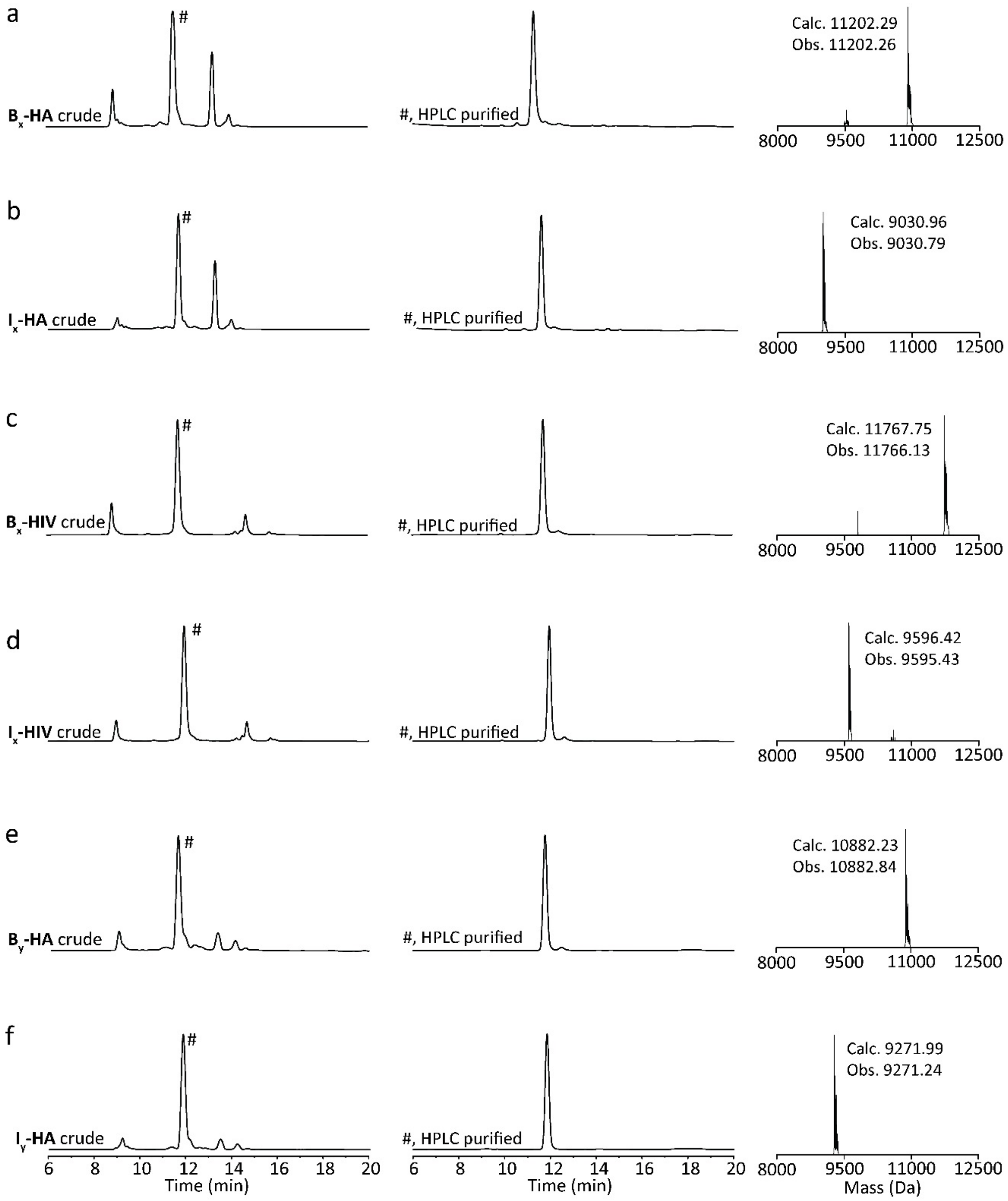

Supplementary Figure 3 | Characterization of POCs. Shown are the HPLC chromatogram for the crude reaction mixture, HPLC chromatogram of HPLC purified POC and deconvoluted mass spectrum of HPLC purified POC (**a**) **B_x-HA**, (**b**) **I_x-HA**, (**c**) **B_x-HIV**, (**d**) **I_x-HIV**, (**e**) **B_y-HA**, (**f**) **I_y-HA**.

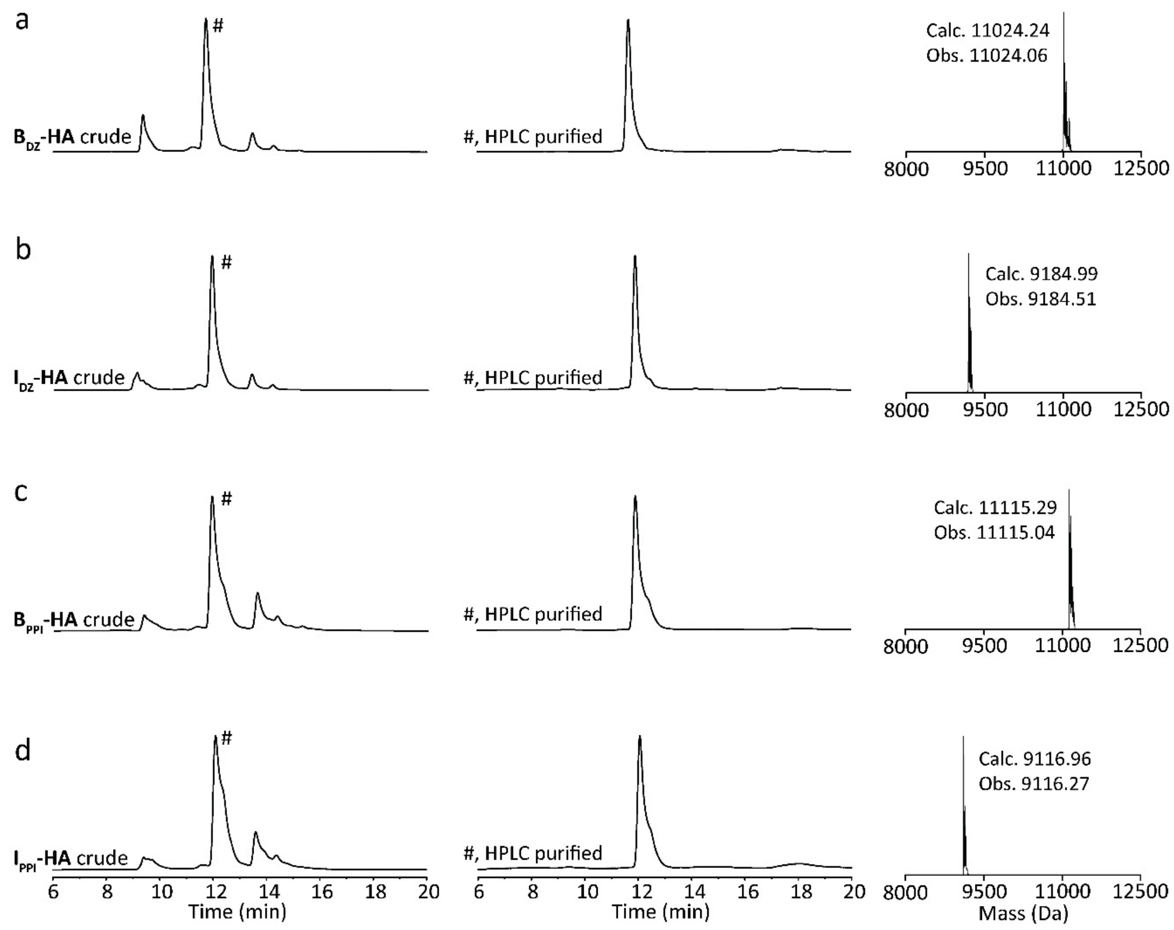

Supplementary Figure 4 | Characterization of POCs. Shown are the HPLC chromatogram for the crude reaction mixture, HPLC chromatogram of HPLC purified POC and deconvoluted mass spectrum of HPLC purified POC (**a**) **B_{Dz}-HA**, (**b**) **I_{Dz}-HA**, (**c**) **B_{PPI}-HA**, (**d**) **I_{PPI}-HA**.

ATSE reaction optimization

For the screening of optimal toehold length **BO** duplexes were created by mixing 10 µL **B_x-HA** or **B_x-HIV** with 10 µL **O_{xn}** (**n** = 0 - 6), 140 µL TE and 40 µL TE / 5xMg^{2+} (TE buffer + 62.5 mM $MgCl_2$), yielding 2.5 µM **BO_{xn}** duplexes in 1x TE/Mg^{2+} and allowed to incubate for 1 h at room temperature. Subsequently, **BO_{xn}** duplexes (5.5 nM), antibody (5 nM) and reporter duplex **Rep** (10 nM) were mixed in 1x TE/Mg^{2+} supplemented with 1 mg mL^{-1} BSA to a total volume of 190 µL and allowed to equilibrate for 1 h at 28 °C. For negative and positive controls the **BO_{xn}** duplex was either omitted or replaced by 5.5 nM of free **O_{x6}**, respectively. Finally, **I_x-HA** or **I_x-HIV** was added to a final concentration of 5 nM and the fluorescence intensity was directly measured on a platereader equilibrated to 28 °C (Infinite F500, Tecan, ex/em = 485/520 nm) with 1 minute intervals for 5 hours. The obtained fluorescence intensities were normalized to the positive and negative controls, resulting in 1 normalized fluorescence unit (n.u.) to correspond to the fluorescence intensity generated by 1 nM **O_{x6}**. Duplicate kinetic traces were averaged and non-linear least square analysis was performed using Supplementary Equation 1, yielding an estimated k_{obs} with standard errors obtain from the Fisher information matrix (Origin 9.1, OriginLab, all R^2 values > 0.9).

$$I = I_0 + A\, e^{-k_{obs}\, t} \quad (1)$$

b. Limit of Detection for the ATSE reaction

To determine the limit of detection (LOD) for the ATSE reaction coupled to the fluorescence readout by the displacement of a reporter duplex by the generated output strand **O**, an antibody titration was performed. To this end, 5.5 nM **BO**, 5 nM **I** and 10 nM **Rep** were mixed with 0 nM to 12.5 nM anti-HA antibody in 1 x TE/Mg^{2+} supplemented with 1 mg mL^{-1} BSA and incubated for 3 hours at 28 °C. Fluorescence intensities were recorded and normalized to a negative control containing no **BO**, and a positive control spiked with 5.5 nM free **O**. This results in 1 normalized unit (n.u.) to correspond to fluorescence generated by 1 nM of **O**. Triplicate measurements were averaged and a linear equation was fitted to the obtained data between 0 nM and 5 nM anti-HA antibody. The LOD was defined by the concentration of anti-HA antibody that corresponds to the fluorescence intensity generated by the blank without antibody + 3 x the standard deviation, yielding a LOD of 23 pM.

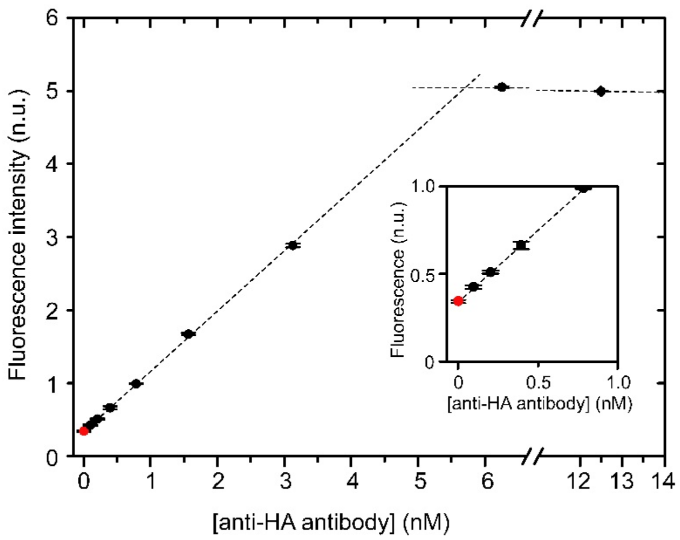

Supplementary Figure 5 | Determination of the limit of detection. Normalized fluorescence intensities generated by the ATSE reaction with [anti-HA antibody] = 0 – 12.5 nM after 3 hours incubation at 28 °C. The limit of detection is defined as the concentration of antibody that generated the same fluorescence as the blank without antibody + 3 x the standard deviation of the blank. [**BO**] = 5.5 nM, [**I**] = 5 nM and [**Rep**] = 10 nM. Error bars represent the standard deviation of triplicate measurements.

Mutiplexed antibody detection

BO duplexes were created in a similar way as described for the toehold screening by hybridizing **B_x-HIV** with **O_{x3}** and **B_y-HA** with **O_{y3}** (Supplementary Table 4). Reporter duplexes were created as described above by thermally annealing **F_x** with **Q_x** and **F_y** with **Q_y**, respectively yielding **Rep_x** and **Rep_y**. Subsequently, **BO** duplexes (5.5 nM each), **I_x-HIV** (5 nM), **I_y-HA** (5 nM), **Rep_x** (1 nM) and **Rep_y** (1 nM) were mixed in 1 x TE/Mg^{2+} supplemented with 1 mg mL^{-1} BSA to a total volume of 180 µL. To this mixture 10 µL buffer or anti-HA antibody and 10 µL buffer or anti-HIV1-p17 antibody was added, yielding all possible combinations of antibody in the same final volume of 200 µL. Directly after the addition of antibodies, fluorescence intensities were measured on a platereader (Saffire-II, Tecan, ex/em_{FAM} = 495/520 nm, ex/em_{TEX} = 595/620 nm) equilibrated to 28 °C with 3 minute intervals for 3 hours. Triplicate measurements were averaged and subsequently the fluorescence intensities after 3 hours incubation was subtracted by the initial (t = 0 minutes) fluorescence intensity of the samples without antibodies and normalized to the samples showing the highest fluorescence intensity after 3 hours incubation. A fixed threshold value of 0.5 n.u. was used to determine true / false values.

Logic operations

BO duplexes were created similar as described above by hybridizing the appropriate combination of oligonucleotides and POCs as indicated in Supplementary Table 4. Subsequently, **BO** duplexes (5.5 nM each) were mixed with the appropriate invading strands (**I**, 5 nM each) and reporter duplex(es) (**Rep**, 1 nM each) in 1 x TE/Mg^{2+} supplemented with 1 mg mL^{-1} BSA to a total volume of 180 µL. To this mixture 10 µL of buffer or anti-HA antibody and 10 µL buffer or anti-HIV1-p17 antibody was added to a final concentration of 10 nM, yielding all possible combinations of antibody in the same final volume of 200 µL. Directly after the addition of antibodies, fluorescence intensities were measured on a platereader (Infinite F500, Tecan, ex/em = 485/520 nm for OR, NOR and AND gate, ex/em = 612/670 nm for NAND gate) equilibrated to 28 °C with 1 minute intervals for 3 hours. For OR and AND gates, the fluorescence intensities after 3 hours incubation was subtracted by the initial (t = 0 minutes) fluorescence intensity of the samples without antibodies and normalized to the samples showing the highest fluorescence intensity at 3 hours incubation. For NOR and NAND gates, the fluorescence intensity after 3 hours incubation was subtracted by a positive control, where **BO** was substituted by free **O**, and normalized to the samples showing the highest observed fluorescence intensity after 3 hours incubation. A fixed threshold value of 0.5 n.u. was used for all experiments to define true / false values. All experiments were performed in *triplo*. Notably, since internal FAM fluorophores are prone to N-quenching by opposite and neighboring nucleotides, the NAND gate was constructed using Cy5 as a fluorophore instead of FAM.[2]

Supplementary Table 4 | Oligonucleotides / POCs used for the construction of DNA-based molecular computing operations.

	Logic Operator				
	**Multiplex**	**OR**	**NOR**	**AND**	**NAND**
**BO**	B_x-HIV + O_{x3}<br>B_y-HA + O_{y3}	B_x-HIV + O_{x3}<br>B_x-HA + O_{x3}	B_x-HIV + $O_{x\text{-}NOR}$<br>B_x-HA + $O_{x\text{-}NOR}$	B_x-HIV + O_{x3}<br>B_y-HA + O_{y3}	B_x-HIV + O_{x3}<br>B_y-HA + $O_{y\text{-}NAND}$
**I**	I_x-HIV<br>I_y-HA	I_x-HIV<br>I_x-HA	I_x-HIV<br>I_x-HA	I_x-HIV<br>I_y-HA	I_x-HIV<br>I_y-HA
**Rep**	$F_x + Q_x$<br>$F_y + Q_y$	$F_x + Q_x$	$F_{NOR} + H_{NOR}$	$F_{AND} + Q_{AND} + H_{x\text{-}(N)AND}$	$F_{NAND} + H_{y\text{-}NAND} + H_{x\text{-}(N)AND}$

Downstream actuation of DNAzyme activity

The **BO** duplex was created similar as described above by hybridizing **B_{Dz}-HA** with **O_{Dz}**. The DNAzyme-Inhibitor duplex (**DzInh**) was created by thermal annealing of **Dz** with **Inh**. Subsequently, **BO** duplex (5nM) was mixed with the invading strand (**I_{Dz}**, 5 nM), **DzInh** duplex (5 nM) and substrate strand (**S**, 10 nM) in 1 x TE/Mg^{2+} supplemented with 1 mg mL^{-1} BSA to a total volume of 190 µL. To this mixture either 10 µL of buffer or anti-HA antibody was added to a final concentration of 5 nM. Directly after addition of the antibody, substrate turnover was monitored by measuring donor (FAM) and acceptor (TAMRA) emission on a platereader (Infinite F500, Tecan, ex_{FAM}/em_{FAM} = 485/520 nm and ex_{FAM}/em_{TAMRA} = 485/590 nm) equilibrated to 28 °C with 1 minute intervals for 3 hours. The DNAzyme activity after incubating for 1 hour in presence and absence of antibody was obtained by deriving the slope of the emission ratio at t = 1 h, yielding a ~20-fold increase in DNAzyme activity when antibody triggered compared to background activation.

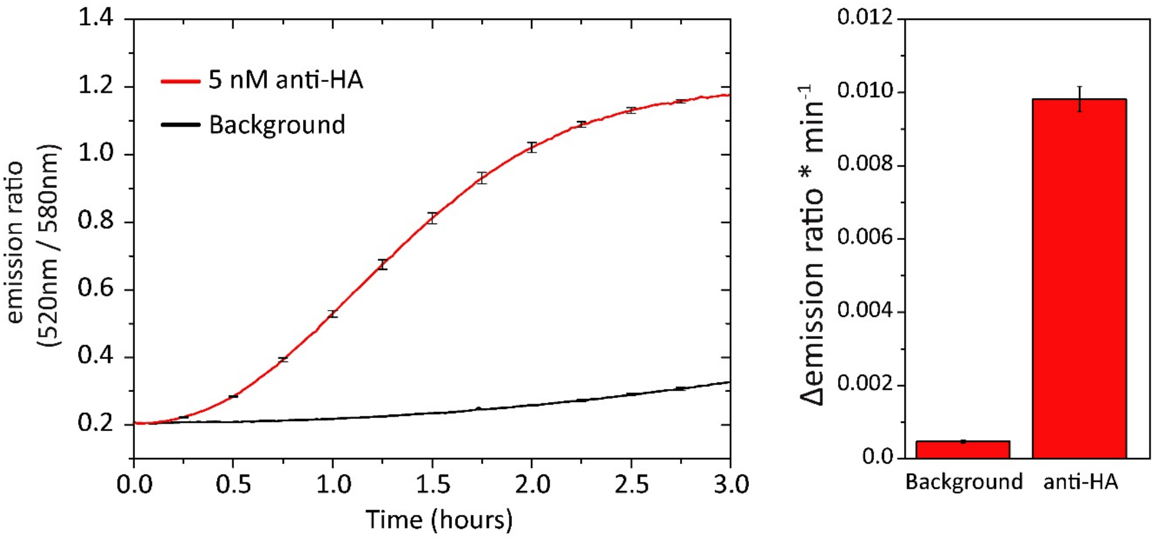

Supplementary Figure 6 | Downstream activation of a catalytic DNAzyme. Average emission ratio (520 nm / 580 nm, ex = 485 nm) in time for the anti-HA antibody triggered circuit (red) and background (black). [**BO**] = [**I**] = [**DzInh**] = [**Ab**] = 5 nM and [**S**] = 10 nM. Error bars represent the standard deviation of triplicate measurements.

Downstream actuation of DNA-directed reporter enzyme

Protein-oligonucleotide conjugates were synthesized and purified as previously reported.[3] In short, amine-modified oligonucleotides (**β-LacE104D** and **BLIP**) were reacted with Sulfo-SMCC to functionalize them with a thiol-reactive maleimide. Subsequently, the oligonucleotides were conjugated site-specifically to an inserted cysteine in the proteins (TEM1-β-lactamaseE104D and BLIP, respectively). Pure protein-oligonucleotide conjugates were obtained by nickel-affinity chromatography followed by anion exchange chromatography. The enzyme-inhibitor complex was formed by mixing **β-LacE104D** (2.5 μM), **BLIP** (5 μM) and template $\mathbf{T_{PPI}}$ (3 μM) in 1 x TE/Mg^{2+} supplemented with 1 mg mL^{-1} BSA and incubated for 1 hour at room temperature. The **BO** duplex was created similar as described above by hybridizing $\mathbf{B_{PPI}}$**-HA** with $\mathbf{O_{PPI}}$. Subsequently, **BO** duplex (5 nM) was mixed with the invading strand ($\mathbf{I_{PPI}}$, 5 nM) and **enzyme-inhibitor** complex (1 nM) in 1 x TE/Mg^{2+} supplemented with 1 mg mL^{-1} BSA to a total volume of 190 μL. To this mixture either 10 μL of buffer or anti-HA antibody was added to a final concentration of 5 nM and allowed to incubate for 3 hours at 28 °C. After 3 hours incubation, 10 μL substrate (CCF2-FA, Invitrogen) was added to a final concentration of 2 μM and fluorescence intensity was directly monitored on a platereader (Saffire-II, Tecan, ex/em = 410/447 nm) equilibrated to 28 °C with 15 second intervals for 30 minutes. Hydrolysis rates were obtained by deriving the slope of fluorescence intensity as a function of time, yielding a 3 fold increase in enzyme activity in the presence of antibody compared to background activity.

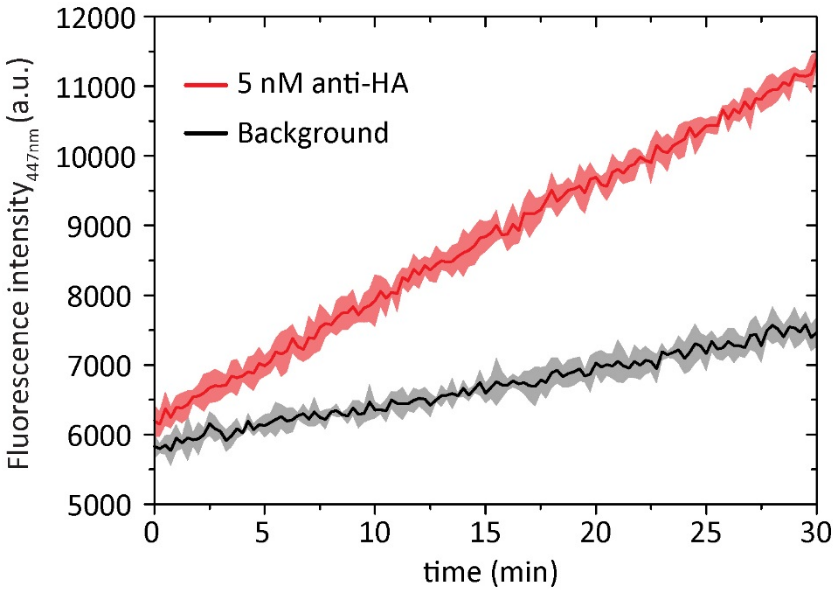

Supplementary Figure 7 | Downstream activation of an enzyme-inhibitor complex. Average fluorescence intensity (447 nm, ex = 410 nm) in time for the anti-HA antibody triggered circuit (red) and background (black). [**BO**] = [**I**] = [**Ab**] = 5 nM and [**enzyme-inhibitor**] = 1 nM. Shaded areas represent the standard deviation of triplicate measurements.

Mechanistic kinetic reaction model

A mathematical model that describes the ATSE reaction was developed using Ordinary Differential Equations (ODE's). Supplementary Figure 8 shows the reactions on which the ODE model is based starting from two different peptide-DNA conjugates (**BO** and **I**) carrying the same peptide-epitope and a bivalent antibody (**Ab**). Binding of the peptide-DNA conjugates to the antibody results in intramolecular strand displacement and the release of DNA strand **O**. The released DNA strand serves as an input to downstream DNA-based molecular circuits. Here, a partial reporter duplex **F·Q** (**Rep**) with a fluorophore-quencher pair is described which releases fluorescent oligonucleotide **F** as a result of strand displacement by output strand **O**. The ODE model was used to compute the heat maps and simulate the traces of the species as a function of time shown in the main text (Fig. 2).

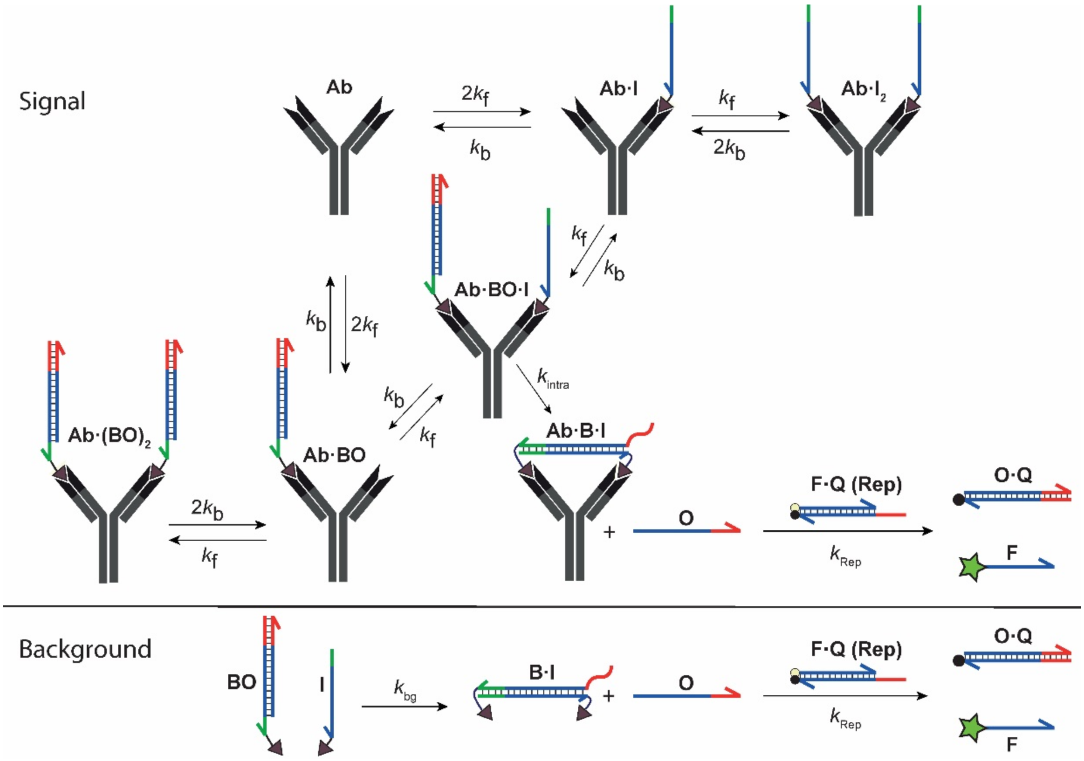

Supplementary Figure 8 | Overview of reactions in the theoretical model in the presence of antibody (signal) and absence of antibody (background).

To obtain an accurate model, most of the kinetic parameters were determined in separate experiments (*vide infra*), including the association rate constant (k_f) for the binding of peptide-DNA conjugates to the anti-HA antibody, the forward rate of the background reaction (k_{bg}) and the forward rate constant (k_{rep}) of the toehold-mediated strand displacement of the reporter complex **F·Q** with ssDNA **O**. The dissociation rate constant k_b was calculated from the association rate constant (k_a) and the dissociation equilibrium constant of peptide-DNA conjugates with the anti-HA antibody (K_d), which was also obtained independently.

Determination of dissociation constant for peptide-epitope binding to the antibody (K_d)

To determine the dissociation equilibrium constant of the HA-peptide-epitope binding to the anti-HA antibody, the C-terminal cysteine of the peptide-epitope was functionalized with a maleimide-activated fluorescein. Binding of the fluorescently labeled peptide-epitope to the anti-HA antibody results in an increased fluorescence polarization. To this end, 0.1 pM – 500 nM of anti-HA antibody was mixed with 2 nM of fluorescently labeled peptide-epitope in 1x TE/Mg^{2+} supplemented with 1 mg mL^{-1} BSA in a final volume of 50 µL and incubated for 1 hour at room temperature. Subsequently, fluorescence polarization was recorded of triplicate individual measurements. To determine whether DNA conjugation has a significant effect on the binding affinity of the peptide to the antibody the fluorescein-labeled peptide was substituted for a peptide-DNA conjugate that was prehybridized to a fluorescein labeled complementary oligonucleotide. Non-linear least square optimization of equation S2 to the mean of the triplicate measurements was performed taking the standard deviation as a weight factor, yielding the dissociation constant K_d = 0.269 ± 0.0195 nM and K_d = 0.775 ± 0.0314 nM for the fluorescein-labelled peptide and peptide-DNA conjugate, respectively.

$$A = A_f + (A_b - A_f) * \frac{([P]+K_d+[Ab]) - \sqrt{([P]+K_d+[Ab])^2 - 4[P][Ab]}}{2[P]}, \quad (2)$$

In Supplementary Equation 2, A is the measured polarization, A_f the polarization coefficient of the free peptide, A_b the polarization coefficient of the bound peptide, $[P]$ the peptide epitope concentration (fixed to 2 nM) and $[Ab]$ the concentration of antigen binding domains (equals two times the antibody concentration).

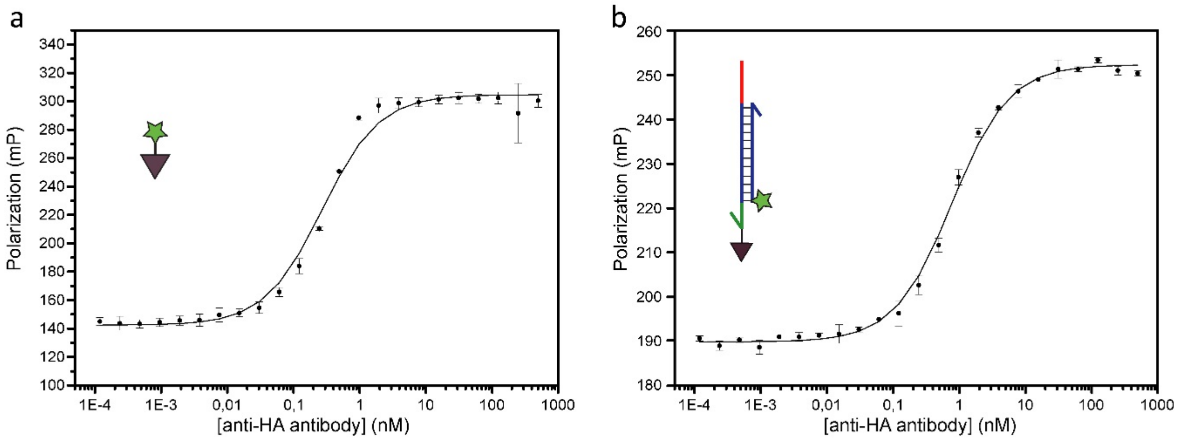

Supplementary Figure 9 | Determination of the dissociation equilibrium constant of HA peptide-epitope binding to the anti-HA antibody. Titration of 0.1 pM – 500 nM antibody to 2 nM of fluorescently labeled peptide-epitope or peptide-DNA conjugate hybridized to a fluorescein-labeled complementary oligonucleotide. The dissociation equilibrium constant was derived by non-linear least square optimization of Supplementary Equation 2 to the mean of triplicate measurements. Error bars represent the standard deviation of triplicate measurements.

To determine the dissociation equilibrium constant of the HIV-peptide-epitope binding to the anti-HIV antibody, the C-terminal cysteine of the peptide-epitope was functionalized with a maleimide-activated fluorescein. Similarly, 2 pM – 1 µM of anti-HIV antibody was mixed with 2 nM of fluorescently labeled peptide-epitope in 1x TE/Mg^{2+} supplemented with 1 mg mL^{-1} BSA in a final volume of 50 µL and incubated for 1 hour at room temperature. Subsequently, fluorescence polarization was recorded of triplicate individual measurements. To determine whether DNA conjugation has a significant effect on the binding affinity of the HIV-peptide to the anti-HIV antibody the fluorescein-labeled peptide was substituted for a peptide-DNA conjugate that was prehybridized to a fluorescein labeled complementary oligonucleotide. Non-linear least square optimization of Supplementary Equation 2 to the mean of the triplicate measurements was performed taking the standard deviation as a weight factor, yielding the dissociation constant K_d = 20.52 ± 1.96 nM and K_d = 15.61 ± 2.04 nM for the fluorescein-labelled peptide and peptide-DNA conjugate, respectively.

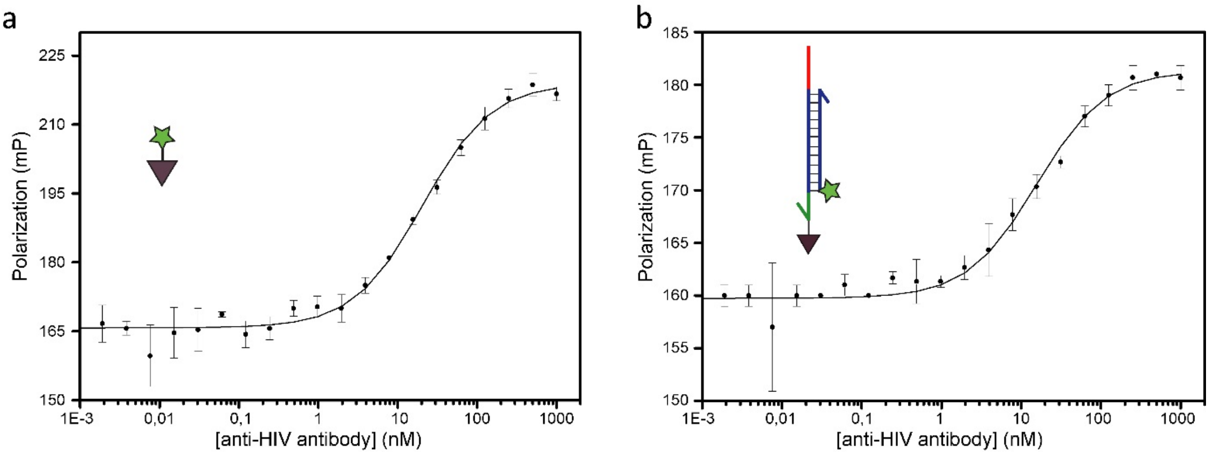

Supplementary Figure 10 | Determination of the dissociation equilibrium constant of HIV peptide-epitope binding to the anti-HIV antibody. Titration of 2 pM – 1 µM antibody to 2 nM of fluorescently labeled peptide-epitope or peptide-DNA conjugate hybridized to a fluorescein-labeled complementary oligonucleotide. The dissociation equilibrium constant was derived by non-linear least square optimization of Supplementary equation 2 to the mean of triplicate measurements. Error bars represent the standard deviation of triplicate measurements.

Determination of the association rate constant for peptide-epitope binding to the antibody (k_f)

The kinetics of binding of a fluorescently labeled peptide-epitope to a bivalent anti-HA antibody was measured using a stopped-flow setup with a dead time of 0.25 ms (BioLogic, MOS-500 spectrophotometer in anisotropy mode, equipped with a SFM-2000 mixing system and 495 nm cut-off filter). Fluorescence anisotropy was recorded over time after mixing the anti-HA antibody with the fluorescently labelled peptide-epitope in 1 x TE/Mg^{2+} + 1 mg mL^{-1} BSA, yielding a final antibody concentration of 2 nM and 2, 10 or 100 nM of the peptide-epitope. For each concentration of peptide-epitope multiple experiments (>6) were performed. Raw data of the experiments was converted to concentration by subtracting the fluorescence anisotropy value at time = 0 and assuming the reaction reached equilibrium within 125 seconds. Furthermore, steady-state concentrations were calculated by the known concentrations of antibody and peptide-epitope and the experimentally determined dissociation constant (K_d). Finally, the mean of the multiple experiments was taken for further analysis.

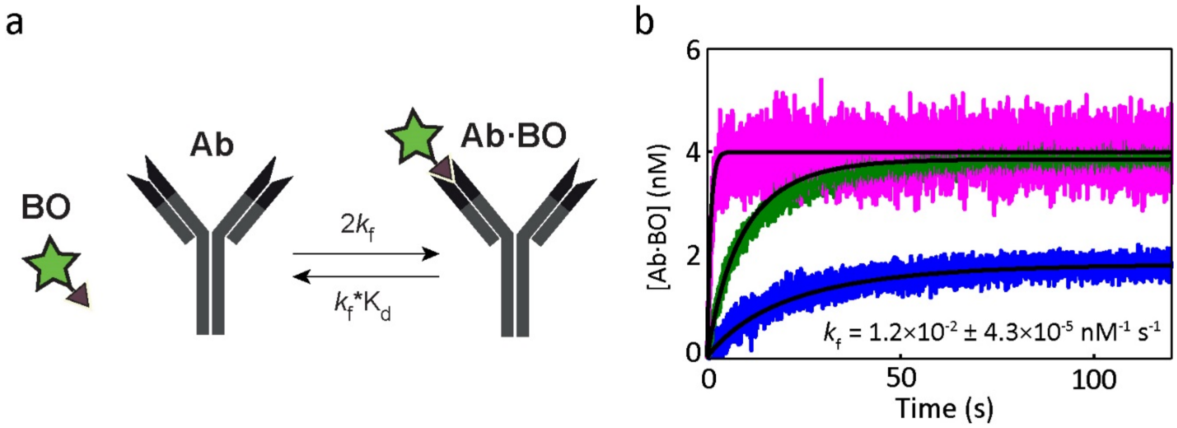

Supplementary Figure 11 | Kinetic characterization of binding of fluorescently-labelled peptide-epitope to a bivalent anti-HA antibody. a, Schematic illustration of the binding of monovalent ligand to bivalent antibody. **b,** Results of non-linear least-square optimization of multiple datasets to the ODE model depicted in Supplementary Equation 3 - 5. 2 nM of antibody was mixed with 2 nM (blue), 10 nM (green) and 100 nM (mangenta) of fluorescently labelled peptide-epitope.

$$\frac{d[Ab]}{dt} = k_f K_d [Ab \cdot BO] - k_f 2[Ab][BO] \quad (3)$$

$$\frac{d[BO]}{dt} = k_f K_d [Ab \cdot BO] - k_f 2[Ab][BO] \quad (4)$$

$$\frac{d[Ab{\cdot}BO]}{dt} = -k_f K_d [Ab \cdot BO] + k_f 2[Ab][BO] \quad (5)$$

An ODE model was used to characterize the kinetics of the peptide-epitope binding to the antibody. The dissociation equilibrium constant (K_d) was determined experimentally (Supplementary Figure 9). Furthermore, the ligand can bind independently at two positions of the antibody. Taking this into account, the antibody concentration in the mathematical model is twice the experimental antibody

concentration (hence the factor of two in front of [*Ab*] in Supplementary Equation 3, 4 and 5). An association rate constant (k_f) of $1.2 \times 10^{-2} \pm 4.3 \times 10^{-5}$ nM^{-1} s^{-1} was obtained by a global non-linear least squares optimization of the three kinetic curves to the ODE model, performed using the Matlab routine *lsqnonlin* employing a subspace trust-region method based on the interior-reflective Newton method. The lower bounds of the 95% confidence intervals and asymptotic standard errors were determined using the observed Fisher information matrix.[4]

Determination of the rate constant for TMSD of the reporter duplex (k_{rep})

To characterize the kinetics of the reporter duplex **F·Q** (**Rep**), experiments were performed using the stopped-flow device in fluorescence mode. Fluorescence intensity was recorded over time after mixing reporter duplex **F·Q**, with output strand **O** in 1 x TE/Mg^{2+} + 1 mg mL^{-1} BSA, yielding a final **F·Q** concentration of 10 nM and 2, 20 or 100 nM of output **O**. For each concentration, multiple experiments (>6) were performed. The raw data was subtracted by the fluorescence at time = 0. Thereafter, the fluorescence was converted to concentration of free **F** by assuming the reaction was equilibrated in 400 seconds. Finally, the mean of the multiple experiments was determined for further analysis.

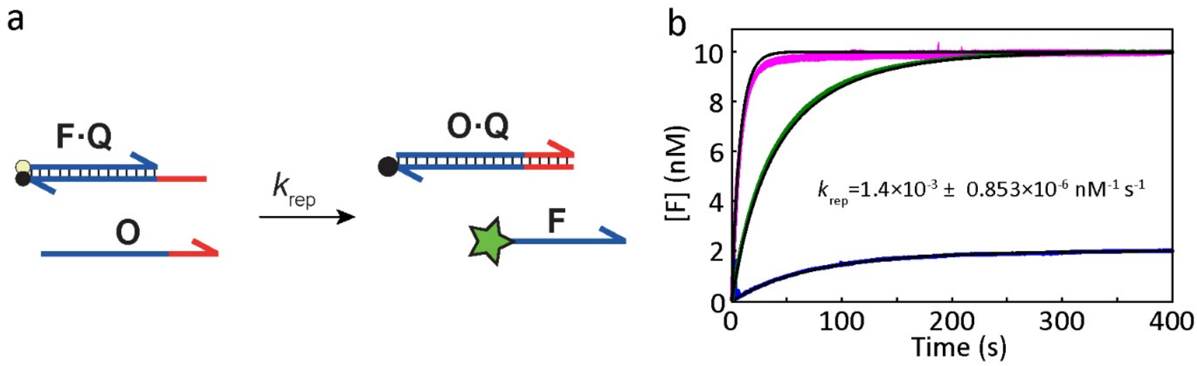

Supplementary Figure 12 | Kinetic characterization of the fluorescent reporter. a, Schematic illustration of the reporter duplex **F·Q** in which the strand **F** is released upon displacement by strand **O**. **b,** Results of non-linear least-square optimization of multiple datasets to the ODE model depicted in Supplementary Equation 6 - 9. 10 nM of reporter duplex **F·Q** was mixed with 2 nM (blue), 20 nM (green) and 100 nM (mangenta) of output **O**.

$$\frac{d[\boldsymbol{O}]}{dt} = -\boldsymbol{k_{rep}}[\boldsymbol{F} \cdot \boldsymbol{Q}][\boldsymbol{O}] \quad (6)$$

$$\frac{d[\boldsymbol{F \cdot Q}]}{dt} = -\boldsymbol{k_{rep}}[\boldsymbol{F} \cdot \boldsymbol{Q}][\boldsymbol{O}] \quad (7)$$

$$\frac{d[O \cdot Q]}{dt} = k_{rep}[F \cdot Q][O] \quad (8)$$

$$\frac{d[\boldsymbol{F}]}{dt} = \boldsymbol{k_{rep}}[\boldsymbol{F} \cdot \boldsymbol{Q}][\boldsymbol{O}] \quad (9)$$

A mathematical model, based on the bimolecular reaction model of DNA strand displacement was used to characterize the kinetics of the reaction.[5] To obtain the second-order rate constant (k_{rep}) non-linear least-square optimization of multiple kinetic traces to the ODE model depicted in

Supplementary Equation 6 - 9 was performed using the Matlab routine lsqnonlin with a subspace trust-region method based on the interior-reflective Newton method, yielding a k_{rep} of 1.4 x 10^{-3} ± 0.853 x 10^{-6} nM^{-1} s^{-1}. To prevent entrapment in local minima of the cost function, Latin Hypercube sampling was used to create twenty initial parameter vectors, with values in between an interval of 0.01 and 100 times of the expected parameter value. From these twenty initial parameter values the parameter values corresponding to the lowest residual sum of squares were selected. The lower bounds of the 95% confidence intervals and asymptotic standard errors were determined using the observed Fisher information matrix.[4]

Determination of the forward rate constant of the background reaction (k_{bg})

To determine the forward rate constant (k_{bg}) of the background reaction the experiment as shown in Supplementary Figure 13 was performed. For each toehold length the fluorescence intensity was measurement in time in duplo and the mean was used for further analysis (Supplementary Figure 14)

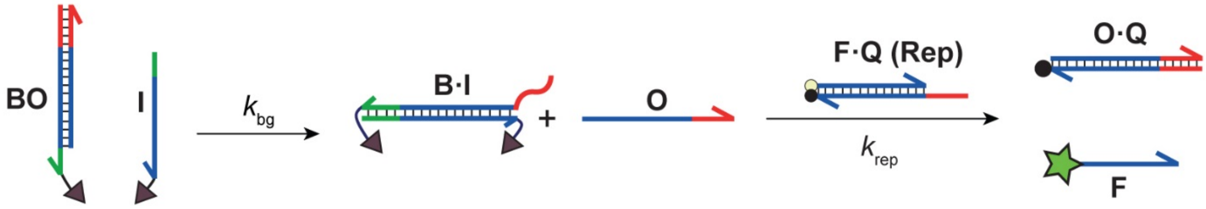

Supplementary Figure 13 | Schematic representation of the experiment performed to determine the forward rate constant of the background reaction. Peptide-DNA conjugate **I** can bind to a toehold on duplex **BO** and displace strand **O**. This reaction is assumed to be irreversible. DNA strand **O** can bind to reporter duplex **F·Q** and displace strand **F**, resulting in an increase in fluorescence.

First, the raw data was subtracted by a negative control where the **BO** duplexes were omitted. Thereafter, the fluorescence was converted to concentration of free oligonucleotide **F** using a control experiment in which 5.5 nM of **O** was mixed with 10 nM of **F·Q**. The fluorescence after completion of this reaction was used to calculate the conversion factor for fluorescence intensity to concentration free **F**.

A kinetic model was developed to characterize the kinetics of the background reaction (Supplementary Table 5). To obtain the forward rate constant of the background reaction (k_{bg}) as function of toehold length non-linear least squares optimization of the ODE model to the experimental data depicted in Supplementary Figure 14 was performed with k_{rep} fixed to its experimentally determined value, using the Matlab routine *lsqnonlin* with a subspace trust-region method based on the interior-reflective Newton method. The lower bounds of the 95% confidence intervals and asymptotic standard errors were determined using the observed Fisher information matrix.[4] Individual rate constants were obtained for the different toehold lengths (Supplementary Table 9).

Supplementary Table 5 | ODE's of the theoretical model of the background reaction in absence of antibody.

Equation number	Species	Differential Equation
10	*[BO]*	$\frac{d[BO]}{dt} = -k_{bg}[BO][I]$
11	*[I]*	$\frac{d[I]}{dt} = -k_{bg}[BO][I]$
12	*[O]*	$\frac{d[O]}{dt} = k_{bg}[BO][I] - k_{rep}[F \cdot Q][O]$
13	*[B · I]*	$\frac{d[B \cdot I]}{dt} = k_{bg}[BO][I]$
14	*[F · Q]*	$\frac{d[F \cdot Q]}{dt} = -k_{rep}[F \cdot Q][O]$
15	*[O · Q]*	$\frac{d[O \cdot Q]}{dt} = k_{rep}[F \cdot Q][O]$
16	*[F]*	$\frac{d[F]}{dt} = k_{rep}[F \cdot Q][O]$

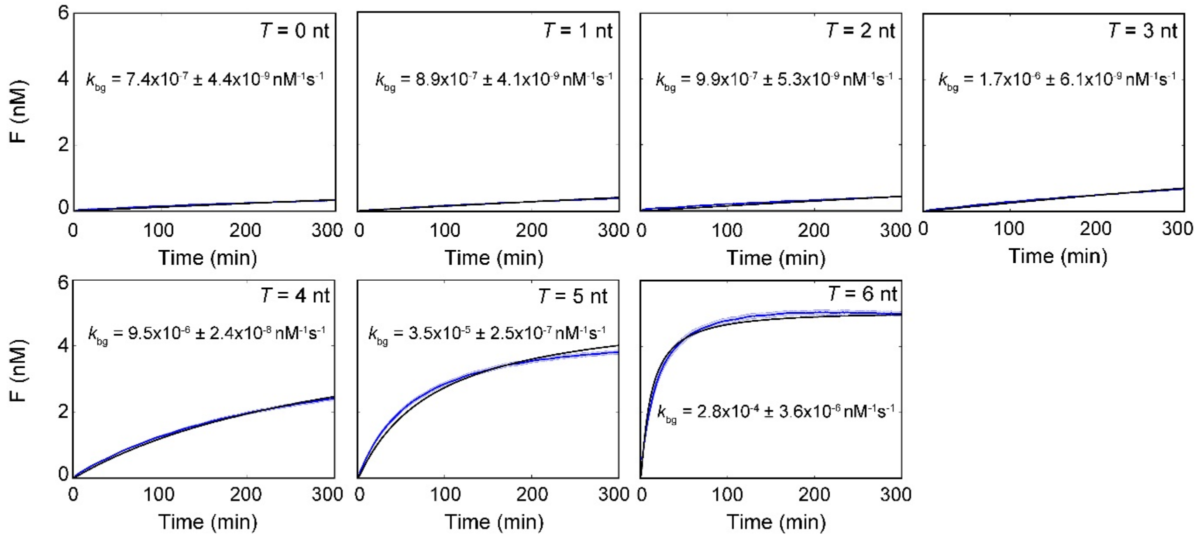

Supplementary Figure 14 | Background strand exchange reaction (blue) for the ATSE reaction components in the absence of antibody for a range of toehold lengths. The shaded area represents the standard error of the mean of duplicate experiments. Non-linear least-squares optimization to the ODE model depicted in Supplementary Table 5 was performed (black) to obtain the forward rate constant of the background reaction (k_{bg}). Experiments were performed with [**BO**] = 5.5 nM, [**I**] = 5 nM and [**F·Q**] = 10 nM.

Determination of the intramolecular rate constant of cyclization of bivalent antibody with Peptide-DNA conjugates (k_{intra})

A mathematical model that describes the complete system was developed using Ordinary Differential Equations (ODE's) with experimentally obtained rate constants (vide supra). The set of ODEs describing the dynamics of the system was obtained by deriving an equation for each of the species in the network using mass-action kinetics. These equations were implemented in MATLAB and solved numerically in time, resulting in a temporal evolution of all species in the system. The differential equations of the theoretical model were deduced from the reaction mechanisms shown in Supplementary Figure 8 using the following assumptions:

1. In the mathematical model the binding and unbinding of the peptide-oligonucleotide conjugates to the anti-HA antibody are described by the association and dissociation rate constants k_f and k_b respectively, which are assumed to be the same as the kinetics of binding and unbinding of fluorescently labeled peptide-epitopes to the antibody. Furthermore, a statistical factor is used when a peptide-oligonucleotide conjugate has two possibilities to bind or dissociate from the antibody.
2. The formation of the cyclic bivalent **Ab·B·I** is assumed to be irreversible on the time scale of the experiment. Previous work has shown that the dissociation rate for this very stable bivalent interaction is very low, requiring overnight equilibration for competition experiments.[6]
3. The cyclization of the antibody with the two heterogeneous peptide-DNA conjugates is described using an intramolecular rate constant (k_{intra}).
4. The toehold-mediated strand displacement of **F** from the reporter complex **F·Q** by DNA strand **O** is an irreversible reaction since the **O·Q** complex does not contain a toehold. The reaction is described using the bimolecular approximation developed by Zhang and Winfree.[5]
5. The background reaction as shown in Supplementary Figure 13 is described using the bimolecular approximation and is assumed to be effectively irreversible as DNA strand **O** is sequestered by an excess of reporter complex **F·Q**.
6. Importantly, the background reaction can also take place in the presence of the antibody. Supplementary Figure 15a shows all possible background reactions on the antibody. If this reaction happens it is assumed cyclization takes place, irrespective whether the other antigen binding site is occupied or not. In the mathematical model these multistep reactions are coarse-grained into one step using a single rate constant k_{bg} and assuming that cyclization is relatively fast and irreversible (Supplementary Figure 15b), irrespective whether the other antigen binding site is occupied or not. Notably, this cyclization results from intramolecular binding of a second peptide epitope and is therefore relatively fast, while cyclization in Supplementary Figure 8

results from strand displacement. This background reaction with the peptide-DNA conjugates bound to the antibody effectively results in a decrease in free peptide-DNA conjugates **BO** and **I** and antibody as well as an increase in DNA strand **O** and cyclized complex (**Ab·B·I**). Therefore, these reactions are included in the mathematical model in the ODE's for free peptide-DNA conjugates **BO** and **I** and free **Ab** (Supplementary Table 6, Supplementary Equation 17-19).

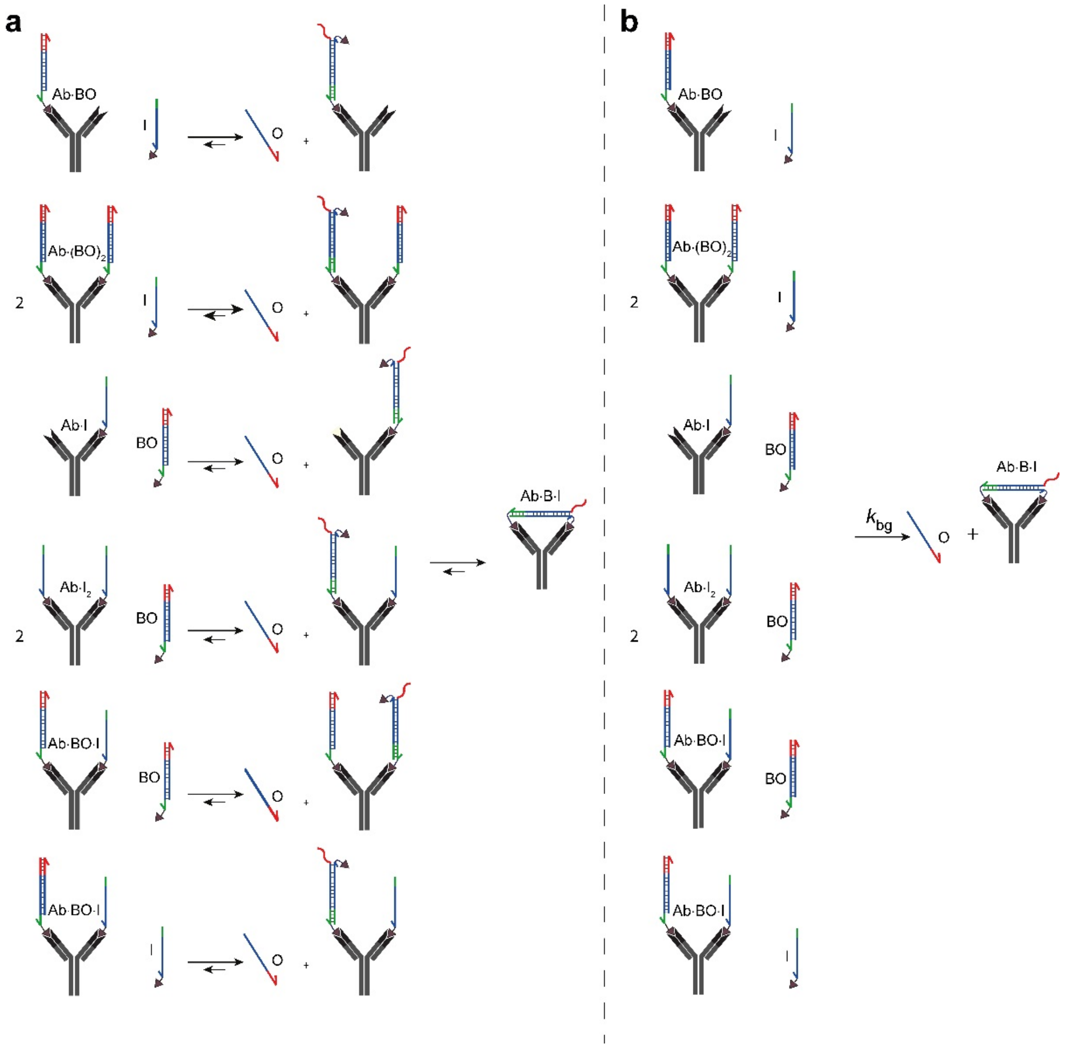

Supplementary Figure 15 | Additional background reactions of various antibody-complexes that were also included in the mathematical model. **a,** Schematic illustration of all possible background reactions at the antibody. A factor of two indicates this reaction has two possibilities to happen. **b,** All these background reactions are included in the mathematical model as a single step with the forward rate constant of the background (k_{bg}) being the rate limiting step.

7. Furthermore, the product of the background reaction [**B·I**], having two epitopes, is likely to bind to the antibody if there is a free antigen binding site available. Therefore, this reaction is also included in the model. Supplementary Figure 16a shows the full reaction scheme, while in the kinetic model we use the simplified scheme in Supplementary Figure 16b which is based on several assumptions. If an epitope of [**B·I**] binds to an antigen binding site of the antibody it is assumed cyclisation, caused by binding of the second epitope, is faster than dissociation of the

peptide-DNA conjugate [**B·I**]. Furthermore, it is assumed this cyclisation is relatively fast and irreversible, irrespective whether the other antigen binding site is occupied or not. Therefore, the reactions in Supplementary Figure 16a are coarse-grained into one step (Supplementary Figure 16b) with the association rate constant of a single peptide-DNA conjugate to the antibody as the rate limiting step. The statistical factor of four is used because two epitopes can bind at two positions of the antibody, while a statistical factor of two is used when one of these positions is occupied.

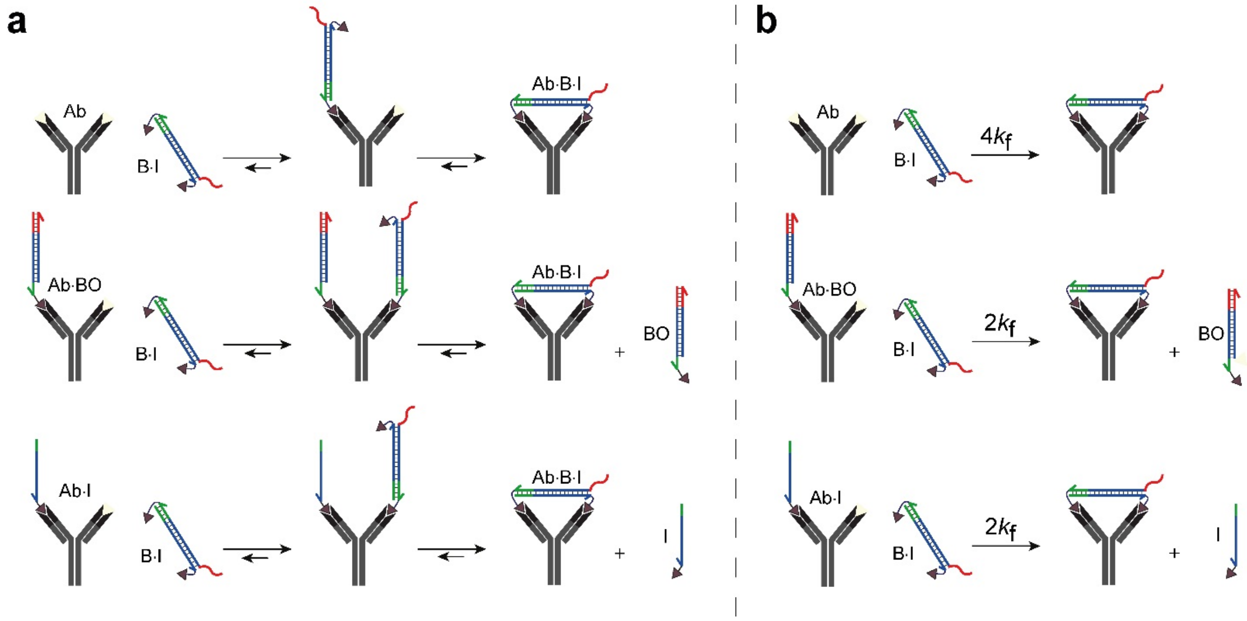

Supplementary Figure 16 | Background reaction product captures antibody. **a,** Illustration of the binding of peptide-DNA conjugate [**B·I**] to the antibody having at least one free antigen binding site, followed by cyclization. **b,** Illustration of the simplified reaction scheme which is used in the kinetic model.

Taking into account all these assumptions the dynamics of the system were obtained by deriving an ODE equation for each of the 14 species (Supplementary Table 6 and 7) in the network using the rate constants in Supplementary Table 9.

Supplementary Table 6 | ODE's of the theoretical model of the ATSE reaction.

Equation number	Species	Differential Equation
17	***[Ab]***	$\frac{d[Ab]}{dt} = k_b[Ab \cdot BO] - 2k_f[Ab][BO] + k_b[Ab \cdot I] - 2k_f[Ab][I]$ $- k_{bg}([Ab \cdot BO][I] + 2[Ab \cdot (BO)_2][I] + [Ab \cdot I][BO] + 2[Ab \cdot I_2][BO] + [Ab \cdot BO \cdot I][BO] + [Ab \cdot BO \cdot I][I])$ $- 4k_f[Ab][B \cdot I]$
18	***[BO]***	$\frac{d[BO]}{dt} = k_b[Ab \cdot BO] - 2k_f[Ab][BO] + 2k_b[Ab \cdot (BO)_2] - k_f[Ab \cdot BO][BO] + k_b[Ab \cdot BO \cdot I] - k_f[Ab \cdot I][BO]$ $- k_{bg}([BO][I] + [Ab \cdot BO][I] + 2[Ab \cdot (BO)_2][I] + [Ab \cdot I][BO] + 2[Ab \cdot I_2][BO] + [Ab \cdot BO \cdot I][BO] + [Ab \cdot BO \cdot I][I])$ $+ 2k_f[Ab \cdot BO][B \cdot I]$
19	***[I]***	$\frac{d[I]}{dt} = k_b[Ab \cdot I] - 2k_f[Ab][I] + 2k_b[Ab \cdot I_2] - k_f[Ab \cdot I][I] + k_b[Ab \cdot BO \cdot I] - k_f[Ab \cdot BO][I]$ $- k_{bg}([BO][I] + [Ab \cdot BO][I] + 2[Ab \cdot (BO)_2][I] + [Ab \cdot I][BO] + 2[Ab \cdot I_2][BO] + [Ab \cdot BO \cdot I][BO] + [Ab \cdot BO \cdot I][I])$ $+ 2k_f[Ab \cdot I][B \cdot I]$
20	***[Ab · BO]***	$\frac{d[Ab \cdot BO]}{dt} = -k_b[Ab \cdot BO] + 2k_f[Ab][BO] + 2k_b[Ab \cdot (BO)_2] - k_f[Ab \cdot BO][BO]$ $+ k_b[Ab \cdot BO \cdot I] - k_f[Ab \cdot BO][I]$
21	***[Ab · I]***	$\frac{d[Ab \cdot I]}{dt} = -k_b[Ab \cdot I] + 2k_f[Ab][I] + 2k_b[Ab \cdot I_2] - k_f[Ab \cdot I][I] +$ $k_b[Ab \cdot BO \cdot I] - k_f[Ab \cdot I][BO]$
22	***[Ab · (BO)₂]***	$\frac{d[Ab \cdot BO_2]}{dt} = -2k_b[Ab \cdot (BO)_2] + k_f[Ab \cdot BO][BO]$
23	***[Ab · I₂]***	$\frac{d[Ab \cdot I_2]}{dt} = -2k_b[Ab \cdot I_2] + k_f[Ab \cdot I][I]$
24	***[Ab · BO · I]***	$\frac{d[Ab \cdot BO \cdot I]}{dt} = -2k_b[Ab \cdot BO \cdot I] + k_f[Ab \cdot BO][I] + k_f[Ab \cdot I][BO] - k_{intra}[Ab \cdot BO \cdot I]$
25	***[Ab · B · I]***	$\frac{d[Ab \cdot B \cdot I]}{dt} = k_{intra}[Ab \cdot BO \cdot I]$ $+ k_{bg}([Ab \cdot BO][I] + 2[Ab \cdot (BO)_2][I] + [Ab \cdot I][BO] + 2[Ab \cdot I_2][BO] + [Ab \cdot BO \cdot I][BO] + [Ab \cdot BO \cdot I][I])$ $+ 4k_f[Ab][B \cdot I] + 2k_f[Ab \cdot BO][B \cdot I] + 2k_f[Ab \cdot I][B \cdot I]$
26	***[O]***	$\frac{d[O]}{dt} = k_{intra}[Ab \cdot BO \cdot I] - k_{rep}[F \cdot Q][O]$ $+ k_{bg}([BO][I] + [Ab \cdot BO][I] + 2[Ab \cdot (BO)_2][I] + [Ab \cdot I][BO] + 2[Ab \cdot I_2][BO] + [Ab \cdot BO \cdot I][BO] + [Ab \cdot BO \cdot I][I])$
27	***[B · I]***	$\frac{d[B \cdot I]}{dt} = k_{bg}[BO][I] - 4k_f[Ab][B \cdot I] - 2k_f[Ab \cdot BO][B \cdot I] - 2k_f[Ab \cdot I][B \cdot I]$
28	***[F · Q]***	$\frac{d[F \cdot Q]}{dt} = -k_{rep}[F \cdot Q][O]$
29	***[O · Q]***	$\frac{d[O \cdot Q]}{dt} = k_{rep}[F \cdot Q][O]$
30	***[F]***	$\frac{d[F]}{dt} = k_{rep}[F \cdot Q][O]$

Supplementary Table 7 | Species in the mathematical model of the ATSE reaction

Species	Description
[Ab]	free concentration of bivalent antibody
[BO]	free concentration of peptide-DNA duplex **BO**
[I]	free concentration of peptide-DNA conjugate **I**
[Ab · BO]	free concentration of antibody · peptide-DNA conjugate **BO** complex
[Ab · I]	free concentration of antibody · peptide-DNA conjugate **I** complex
[Ab · (BO)$_2$]	free concentration of antibody · 2 peptide-DNA conjugate **BO** complex
[Ab · I$_2$]	free concentration of antibody · 2 peptide-DNA conjugate **I** complex
[Ab · BO · I]	free concentration of antibody · peptide-DNA conjugate **BO** · peptide-DNA conjugate **I** complex
[Ab · B · I]	free concentration of cyclized antibody · peptide-DNA conjugate **B** · peptide-DNA conjugate **I** complex
[O]	free concentration of DNA strand ***O***
[B · I]	free concentration of partial duplex **B · I**
[F · Q]	free concentration of partial duplex **F · Q**
[O · Q]	free concentration of duplex **O · Q**
[F]	free concentration of DNA strand **F**

Supplementary Table 8 | Rate constants used for the ATSE reaction model.

Parameter	Description
k_b	Dissociation rate constant (s^{-1}) describing the dissociation of the peptide-DNA conjugates (**BO** and **I**) from antibody (**Ab**).
k_f	Association rate constant (nM^{-1} s^{-1}) for the binding of peptide-DNA conjugates (**BO** and **I**) to antibody (**Ab**).
k_{intra}	association rate constant (s^{-1}) for the cyclization of **Ab · BO · I** complex yielding **Ab · B · I** and free **O**.
k_{rep}	forward rate constant (nM^{-1} s^{-1}) of the second order displacement reaction in which DNA strand **O** binds to partial duplex **F · Q** and displaces DNA strand **F**.
k_{bg}	forward rate constant (nM^{-1} s^{-1}) of the background reaction in which peptide-DNA conjugate **I** binds to peptide-DNA conjugate **BO** and displaces DNA strand **O**.

The only unknown kinetic parameter in the mathematical model is the rate constant of cyclization of bivalent anti-HA antibody with peptide-DNA conjugates (k_{intra}). An estimate of k_{intra} for each toehold length was obtained by performing non-linear least square analysis of the kinetic model using the experimental data of the complete system in presence of anti-HA antibody (Supplementary Figure 17). The raw data of the experiments (performed in duplo) was subtracted by the fluorescence intensity of a control lacking **BO**. Subsequently, the fluorescence was converted to concentration of ssDNA **F** using a control experiment in which 5.5 nM of ssDNA **O** was mixed with 10 nM of reporter duplex (**F·Q**). The fluorescence after completion of the control experiment (subtracted by the fluorescence from the unreacted reporter complex) was used to calculate the conversion factor for fluorescence intensity to concentration of ssDNA **F**. To obtain the intramolecular rate constant of cyclization (k_{intra}) non-linear least squares optimization was performed (Supplementary Figure 17) for each toehold length using the differential equation of Supplementary Table 6 and the Matlab routine *lsqnonlin* with a subspace trust-region method based on the interior-reflective Newton method. The lower bounds of the 95% confidence intervals and asymptotic standard errors were determined using the observed Fisher information matrix.[4] The concentration of antibody used in the non-linear least squares optimization was obtained experimentally from the ATSE reaction with a toehold of 3 nt, where it was assumed that the concentration of antibody was equal to the concentration of fluorescent product **F** after 300 min reaction. This reaction was chosen since the ATSE goes to complete conversion, whereas the contribution of the background reaction remains negligible. For a toehold length of 5 and 6 nt, k_{intra} could not be determined reliably since the ATSE reaction at these toehold lengths is dominated by the background reaction.

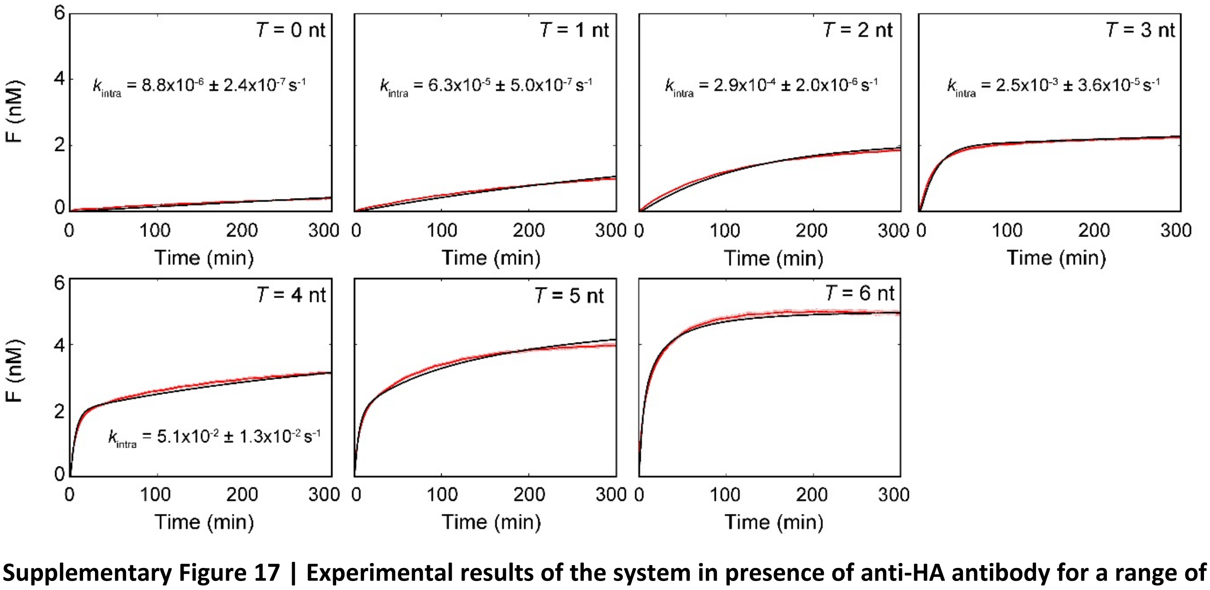

Supplementary Figure 17 | Experimental results of the system in presence of anti-HA antibody for a range of toehold lengths. The shaded area is the standard error of the mean of experiments performed in duplo. Non-linear least-squares optimization was performed (black) to obtain the rate constant of cyclisation via strand displacement (k_{intra}) using the differential equations of Supplementary Table 6. Experiments were performed with [**BO**] = 5.5 nM, [**I**] = 5 nM, [**Ab**] = 2 nM and [<u>**FQ**</u>] = 10 nM.

Supplementary Table 9 | Overview of parameters used in mathematical model for which the values were obtained from non-linear least square analysis of experimental data.

**Parameter**	**Value**						
**K_d (nM)**	0.24						
**k_f (nM^{-1} s^{-1})**	1.2E02						
**k_{rep} (nM^{-1} s^{-1})**	1.4E-3						
	**Toehold length**						
	0	***1***	***2***	***3***	***4***	***5***	***6***
**k_{bg} (nM^{-1} s^{-1})**	7.4E-07 ± 4.4E-09	8.9E-07 ± 4.1E-09	9.9E-07 ± 5.3E-09	1.7E-06 ± 6.1E-09	9.5E-06 ± 2.4E-08	3.5E-05 ± 2.5E-07	2.8E-04 ± 3.6E-06
**k_{intra} (s^{-1})**	8.8E-06 ± 2.4E-07	6.3E-05 ± 5.0E-07	2.9E-04 ± 2.0E-06	2.5E-03 ± 3.6E-05	5.1E-02 ± 1.3E-02	N.D.[a]	N.D.[a]

a Not determined. At a toehold length of 5 and 6 nucleotides the ATSE reaction is dominated by the background reaction. Therefore, one cannot reliably obtain k_{intra}.

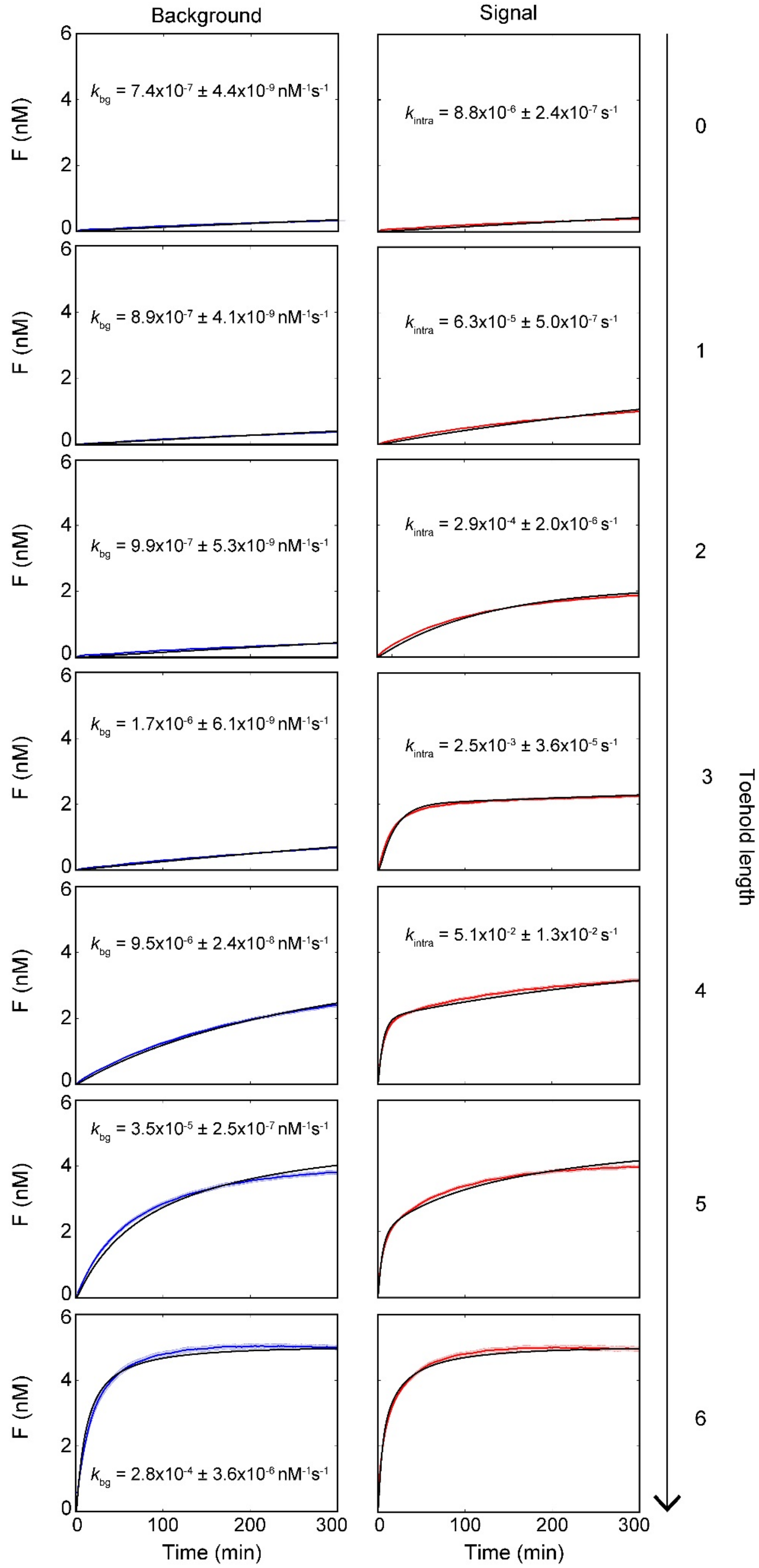

Supplementary Figure S18 | Experimental results of Supplementary Figure 14 (blue) and Supplementary Figure 17 (red) combined. The background of the system in absence of antibody for a range of toehold lengths is shown in blue. The signal of the system in presence of anti-HA antibody for a range of toehold lengths is shown in red. The shaded area is the standard error of the mean of experiments performed in duplo. Non-linear least-squares optimization was performed (black) to obtain the forward rate constant of the background reaction (k_{bg}) and intramolecular rate constant of cyclization (k_{intra}) using the differential equations of Supplementary Table 5 and Supplementary Table 6, respectively. Experiments were performed with [**BO**] = 5.5 nM, [**I**] = 5 nM, [**Ab**] = 2 nM and [**FQ**] = 10 nM.

Simulations

The experimentally obtained kinetic parameters k_b, k_f, k_{rep}, k_{bg} and k_{intra}, were used to simulate the concentration of the individual reaction components as a function of time for the system with the optimal toehold length of 3 nt. In the simulation shown in Supplementary Figure 19, all the components of the ATSE reaction except **I** were premixed and assumed to reach thermodynamic equilibrium before the start of the reaction. At t=0 the ATSE reaction was initiated by the addition of 5 nM **I**. Supplementary Figure 19a shows a decrease in species **I** and an increase in **BO** resulting from the addition of 5 nM **I** to the pre-equilibrated mixture of species **BO**, **Ab** and **F·Q**. This behavior is caused by competition of species **BO** and **I** for the same binding sites of specie **Ab**. Likewise, species **Ab·BO** and **Ab·(BO)$_2$** show a relatively fast initial decrease resulting from equilibration of binding of species **BO**, **I** and **Ab** after addition of species **I**. Next, a more gradual decrease results from cyclization. Furthermore, species **Ab·I**, **Ab·I$_2$** and **Ab·BO·I** show a relatively fast initial increase resulting from equilibrating of binding of species **BO**, **I** and **Ab** after addition of species **I**, followed by a more gradual decrease resulting from intramolecular cyclization. In Supplementary Figure 19c the time traces of the products of cyclization (**Ab·B·I** and **O**) are shown. Furthermore, a relatively fast initial increase in fluorescent DNA strand **F**, resulting from intramolecular cyclization and the background reaction, is followed by a linear increase resulting from a continuing background reaction when the ATSE reaction has reached completion.

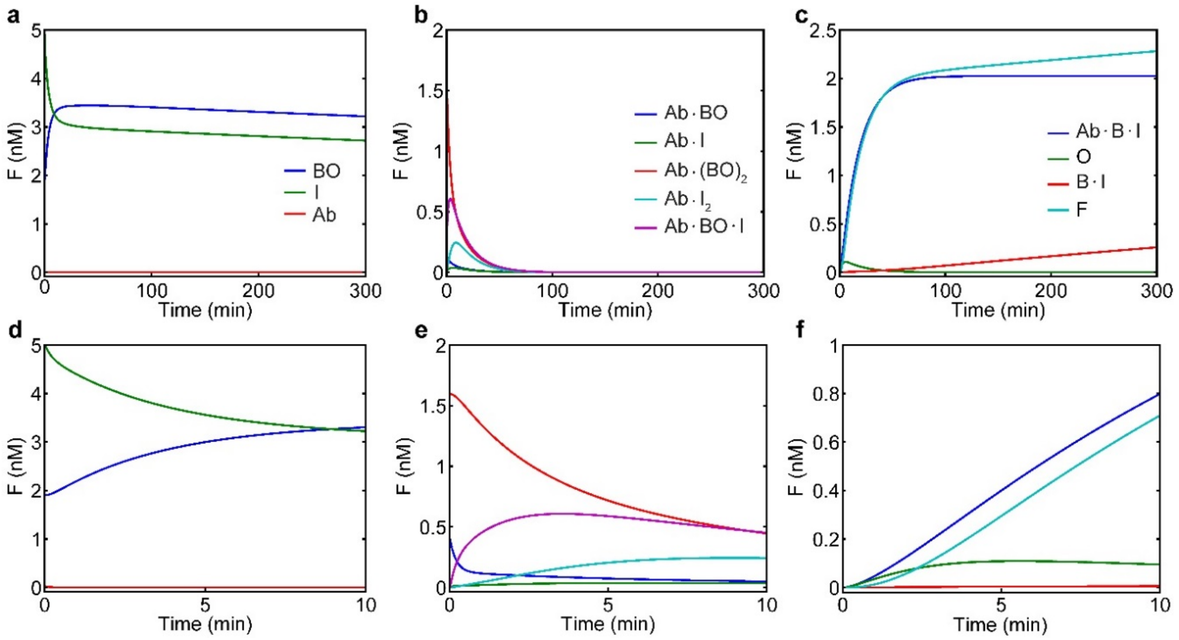

Supplementary Figure 19 | a-c, Simulation of the various species formed during the ATSE reaction. The time traces of the various species were simulated using the ODE model (Supplementary Table 6) with the kinetic parameters (Supplementary Table 9) for a toehold length of 3 nt. The simulations were performed with concentrations of 2 nM, 5.5 nM, 5 nM and 10 nM for the species **Ab, BO**, **I** and probe **F·Q** respectively, with species **BO**, **Ab** and **F·Q** being pre-equilibrated. **d-f,** The time traces of the species for the first ten minutes of the simulation of figures a-c.

Supplementary References

1. Tataurov, A. V., You, Y. & Owczarzy, R. Predicting ultraviolet spectrum of single stranded and double stranded deoxyribonucleic acids. *Biophys. Chem.* **133,** 66–70 (2008).
2. Padirac, A., Fujii, T. & Rondelez, Y. Quencher-free multiplexed monitoring of DNA reaction circuits. *Nucl. Acids Res.* **40,** e118–e118 (2012).
3. Janssen, B. M. G., Engelen, W. & Merkx, M. DNA-directed control of enzyme–inhibitor complex formation: a modular approach to reversibly switch enzyme activity. *ACS Synth. Biol.* **4,** 547–553 (2015).
4. Press, W. H. *Numerical recipes 3rd edition: The art of scientific computing*. (Cambridge University Press, 2007).
5. Zhang, D. Y. & Winfree, E. Control of DNA strand displacement kinetics using toehold exchange. *J. Am. Chem. Soc.* **131,** 17303–17314 (2009).
6. Janssen, B. M. G. *et al.* Reversible blocking of antibodies using bivalent peptide–DNA conjugates allows protease-activatable targeting. *Chem. Sci.* **4,** 1442–1450 (2013).
